# Supplementary material for: Lighter: fast and memory-efficient sequencing error correction without counting
Source: Genome Biol. 2014 Nov 15;15(11):509. doi: 10.1186/s13059-014-0509-9 (PMC4248469; doi:10.1186/s13059-014-0509-9)
Supplement: Supplementary file 1 — Supplementary material for ‘Lighter: fast and memory-efficient error correction without counting’. [file 13059_2014_509_MOESM1_ESM.pdf]

Supplementary material for "Lighter: fast and memory-efficient error correction without counting"

Li Song, Liliana Florea, Ben Langmead

August 6, 2014

Supplementary Note 1: Pattern-blocked Bloom filter

For a standard Bloom filter, each of the h hash functions could map item o to any element of the bit array. The bit array will often be very large, much larger than the processor cache. Thus, each probe into the bit array is likely to cause a cache miss. Putze *et al* [5] propose a *blocked* Bloom filter. Given a block size b, the first hash function $H_0(o)$ is used to select a size-b block of consecutive positions in the bit array. Then, $H_1(o), ..., H_{h-1}(o)$ map o onto elements of that block. When b is less than or equal to the size of a cache line, the h accesses will tend to cause only one or two cache misses, rather than approximately h cache misses.

The drawback is that h and m must be somewhat larger to achieve the same false positive rate (FPR) as a corresponding standard Bloom filter. To estimate the FPR of the blocked Bloom filter, we can consider each of the possible $m - b + 1$ blocks. For the i-th block, the FPR within the block is $(b'_i/b)^h$, where b'_i is the number of bits set to 1 in block i. So the overall FPR is:

$$\frac{\sum_i (b'_i/b)^h}{m - b + 1}$$

Putze *et al* also propose a *pattern-blocked* Bloom filter [5], where the difference is that instead of updating the h positions in the block separately, we pre-compute a list of patterns where each pattern is a bit-mask describing how to update h positions in a block with a few bitwise operations. To perform such an update we first find the appropriate pattern using hash function, then update the corresponding positions simultaneously. In Lighter, 64-bit integers are used to form the mask. For example, if $b = 256$, the pattern is made up of 4 64-bit integers, and we can update in 4 64-bit operations, regardless of h. The FPR formula above still roughly estimates the FPR for the pattern-blocked bloom filter.

Supplementary Note 2: Correcting positions at the ends of reads

If the error is located near the end of the read and some candidate substitutions are equally good, we will extend reads using the k-mer reported in Bloom filter A for each candidates. Lighter extends the read base by base. For the new base beyond the read, Lighter tries all the four nucleotides in the order of "A","C", "G", "T", and uses the first nucleotide creating a k-mer that can be found in Bloom filter A. This procedure is terminated until all the nucleotides fails or the distance to the candidate substitution's position is larger than k - 1. Then we choose the candidate substitution with the longest extension based on this greedy procedure. As a result, we can solve some ties that are more likely to happened near the end of a read due to insufficient extension.

Supplementary Note 3: Scaling with depth of sequencing

Scaling with coverage

Lighter's accuracy is near-constant as the depth of sequencing K increases and its memory footprint is held constant. The basic idea is that as K increases, we adjust α in inverse proportion. That is, we hold αK constant. For concreteness, consider two scenarios: scenario I, where the total number of k-mers is K_1 and subsampling fraction is α_1, and scenario II where the number is $K_2 = zK_1$ and subsampling fraction is $\alpha_2 = \alpha_1/z$.

Contents of Bloom filter A. The occupancy of Bloom filter A, as well as the fraction of correct k-mers in A, are approximately the same in both scenarios. This follows from the fact that $\kappa'_c \sim Pois(\alpha K(1-\epsilon)/G)$, $\kappa'_e \sim Pois(\alpha K\epsilon/H)$, and αK, ϵ, G, and H are constant across scenarios. This is also supported by our experiments, as seen in the main body of the manuscript. Because the occupancy does not change, we can hold the Bloom filter's size constant while achieving the same false positive rate.

Accuracy of trusted / untrusted classifications. Also, if a read position and its neighbors within $k-1$ positions on either side are error-free, then the probability it will be called trusted does not change between scenarios. We mentioned that when α is small, $P(\alpha_1) \approx P(\alpha_1/z) = P(\alpha_2)$. We also showed that the false positive rate of the bloom filter is approximately constant between scenarios, so $P^*(\alpha_1) \approx P^*(\alpha_1/z) = P^*(\alpha_2)$. Thus, the thresholds y_x will also remain unchanged. $p_c = (p(\kappa'_c \geq 1))/(p(\kappa_c \geq 1))$ is the probability a correct k-mer is in the subsample given that it was sequenced. $p_c = (1-e^{-\alpha(1-\epsilon)K/G})/(1-e^{-(1-\epsilon)K/G}) \approx 1-e^{-\alpha(1-\epsilon)K/G}$, since $(1-\epsilon)K/G$ is large. p_c is constant across scenarios since αK, ϵ, and G are constant. Since p_c is constant, the parameters of the $B_{e,x}$ distribution are constant and the probability a correct position will be called trusted is also constant.

Now we consider an incorrect read position. We ignore false positives from Bloom filter A for now. $p_e = p(\kappa'_e \geq 1)/p(\kappa_e \geq 1) = (1-e^{-\alpha\epsilon K/H})/(1-e^{-\epsilon K/H})$ is the probability an incorrect k-mer is in the subsample given that it was sequenced. Since $\epsilon K/H$ is close to 0, $e^{-\epsilon K/H} \approx 1-\epsilon K/H$ and $p_e \approx (\alpha\epsilon K/H)/(\epsilon K/H) = \alpha$. Say an incorrect read position is covered by x k-mers; if $B_{e,x}$ is a random variable for the number of k-mers overlapping the position that appear in Bloom filter A, then $B_{e,x} \sim Binom(x, p_e) \approx Binom(x, \alpha)$. The probability of falsely trusting a position is therefore: $p(B_{e,x} \geq y_x) = \sum_{i=y_x}^{x} \binom{x}{i} p_e^i (1-p_e)^{x-i} \approx \sum_{i=y_x}^{x} \binom{x}{i} \alpha^i (1-\alpha)^{x-i}$. If we omit the $(1-\alpha)^{x-i}$ term in the sum, what remains is an upper bound, i.e. $\sum_{i=y_x}^{x} \binom{x}{i} \alpha^i (1-\alpha)^{x-i} \leq \sum_{i=y_x}^{x} \binom{x}{i} \alpha^i$. Since $\alpha_2 = \alpha_1/z$, the upper bound in scenario II is lower by a factor of at least $1/z$ relative to the upper bound in scenario I. So an upper bound on the probability of labeling an incorrect position as trusted decreases by a factor of at least z. When K increases, the number of distinct test cases for incorrect positions increases by a factor of at most z. Thus, we expect the total number incorrect positions labeled as trusted to remain approximately constant.

When α is small, the false positive rate β may dominate the probability p_e. In practice, however, the false positive rate is usually small enough that the probability of a incorrect position being labeled as trusted due to false positives is extremely low. For example, when k-mer length $k = 17$, the false positive rate of Bloom A ≈ 0.004, the threshold $y_{2k-1} = 6$, and $\alpha = 0.05$. In this situation, $p(B_{e,x} \geq y_x) \approx 5 \cdot 10^{-11}$.

The above is not an exhaustive analysis, since we have not examined the case where a read position is error-free but not all of its neighbors within $k-1$ positions on either side are error-free. In this case, whether the threshold is passed depends chiefly on the whereabouts of the nearby errors.

Contents of Bloom filter B. Given the analysis in the previous section, we expect that the collection of k-mers drawn from the stretches of trusted positions in the reads will not change much across scenarios and, therefore, the contents of Bloom filter B will not change much. This conclusion is also supported by our experiments, as seen in the main body of the manuscript.

Supplementary Note 4: Hardware Environment

This section describes the hardware we used in our experiments.
/proc/meminfo:

```
MemTotal:     528650220 kB
MemFree:        2230712 kB
Buffers:         288652 kB
Cached:       520412244 kB
SwapCached:        3036 kB
Active:         1647888 kB
Inactive:     519194196 kB
HighTotal:            0 kB
HighFree:             0 kB
LowTotal:     528650220 kB
LowFree:        2230712 kB
SwapTotal:      2096472 kB
SwapFree:       2076884 kB
Dirty:                0 kB
Writeback:            0 kB
AnonPages:       140232 kB
Mapped:           30464 kB
Slab:           5148768 kB
PageTables:       13968 kB
NFS_Unstable:         0 kB
Bounce:               0 kB
CommitLimit:  266421580 kB
Committed_AS:    942108 kB
VmallocTotal: 34359738367 kB
VmallocUsed:     282824 kB
VmallocChunk: 34359455519 kB
HugePages_Total:     0
HugePages_Free:      0
HugePages_Rsvd:      0
Hugepagesize:     2048 kB
```

/proc/cpuinfo

```
processor       : 0
vendor_id       : AuthenticAMD
cpu family      : 16
model           : 9
model name      : AMD Opteron(tm) Processor 6172
stepping        : 1
cpu MHz         : 2100.025
cache size      : 512 KB
physical id     : 0
siblings        : 12
core id         : 0
cpu cores       : 12
apicid          : 0
fpu             : yes
```

```
fpu_exception   : yes
cpuid level     : 5
wp              : yes
flags           : fpu vme de pse tsc msr pae mce cx8 apic sep
mtrr pge mca cmov pat pse36 clflush mmx fxsr sse sse2 ht syscall
nx mmxext fxsr_opt pdpe1gb rdtscp lm 3dnowext 3dnow rep_good constant_tsc
nonstop_tsc amd_dcm pni cx16 popcnt lahf_lm cmp_legacy svm extapic
cr8_legacy abm sse4a misalignsse 3dnowprefetch osvw ibs skinit wdt nodeid_msr
bogomips        : 4200.04
TLB size        : 1024 4K pages
clflush size    : 64
cache_alignment : 64
address sizes   : 48 bits physical, 48 bits virtual
power management: ts ttp tm stc 100mhzsteps hwpstate [8]

...
```

The computer we used has 48 such cores.

Supplementary Note 5: Command lines

The commands that we used for all the experiments are available at:
`https://github.com/mourisl/Lighter_paper/blob/revision1/README.md`.

Supplementary Table 1: Quality-free simulation results

Here we give accuracy results from an evaluation similar to that shown in Table 1, except with quality values omitted. Each of the tools was run on a FASTA file (with no qualities) instead of a FASTQ file. Quake and Bless are omitted as they require quality values. The simulated error rate is 1% in these experiments.

Coverage		35×	70×	140×
α for Lighter		0.2	0.1	0.05
Recall	soapec	63.45	63.36	63.07
	musket	93.75	93.73	93.73
	lighter	99.89	99.84	99.88
Prec	soapec	99.99	99.99	99.99
	musket	99.99	99.99	99.99
	lighter	99.96	99.95	99.95
F-score	soapec	77.63	77.57	77.35
	musket	96.77	96.76	96.76
	lighter	99.92	99.90	99.91
Gain	soapec	63.44	63.36	63.06
	musket	93.74	93.72	93.72
	lighter	99.84	99.79	99.82

Supplementary Table 2: Simulation results using the Art simulator.

Here we give accuracy results from an evaluation similar to that shown in Table 1, except that the simulation was conducted using `art_illumina` v2.1.8 from the Art [2] package.

Coverage		35×	70×	140×
α for Lighter		0.2	0.1	0.05
Recall	quake	99.46	99.47	99.46
	soapec	77.97	77.98	78.02
	musket	99.93	99.93	99.93
	bless	99.95	99.94	99.94
	lighter	99.91	99.94	99.94
Prec	quake	99.99	99.99	99.99
	soapec	99.99	100.00	99.99
	musket	99.99	99.99	99.99
	bless	99.57	99.55	99.54
	lighter	99.98	99.99	99.99
F-score	quake	99.73	99.73	99.73
	soapec	87.62	87.63	87.65
	musket	99.96	99.96	99.96
	bless	99.76	99.75	99.74
	lighter	99.95	99.96	99.96
Gain	quake	99.45	99.45	99.45
	soapec	77.97	77.98	78.01
	musket	99.93	99.92	99.93
	bless	99.52	99.49	99.48
	lighter	99.89	99.92	99.92

Supplementary Table 3: Simulation results with *C. elegans* genome.

Accuracy evaluation for the simulated data set from *C. elegans* genome with 35× coverage and 1% error rate using Mason [1] v0.1.2. The row labeled k gives the selected k-mer sizes.

	Quake	SOAPec	Musket	Bless	Lighter
Recall	85.70	53.40	90.31	98.99	98.12
Precision	99.82	99.84	99.59	95.64	99.66
F-score	92.22	69.58	94.72	97.29	98.88
Gain	85.55	53.31	89.94	94.48	97.78
k	19	23	27	31	31

Supplementary Table 4: Alignment statistics for E. coli dataset using –very-fast.

Alignment statistics for the 75× *E. coli* data set using Bowtie 2 [3] v2.2.2 with `--very-fast`. The column labeled k gives the selected k-mer sizes.

		Read Level		Base Level	
	k	Mapped Reads	Increase (%)	Matches/aligned base (%)	Increase (%)
Original	-	3,459,316	-	99.048	-
Quake	19	3,373,491	-2.48	99.659	0.62
SOAPec	17	3,464,456	0.15	99.135	0.09
Musket	17	3,467,805	0.25	99.601	0.56
Bless	19	3,468,604	0.27	99.667	0.62
Lighter	19	3,478,210	0.55	99.641	0.60

Supplementary Table 5: Alignment statistics for E. coli dataset using BWA-MEM.

Alignment statistics for the 75× *E. coli* data set using BWA-MEM [4] v0.7.9a-r786 with default parameters.

		Read Level		Base Level	
	k	Mapped Reads	Increase (%)	Matches/aligned base (%)	Increase (%)
Original	-	3,482,421	-	98.936	-
Quake	19	3,373,877	-3.12	99.968	1.04
SOAPec	17	3,482,422	0.00	99.039	0.10
Musket	17	3,482,735	0.01	99.599	0.67
Bless	19	3,481,889	-0.02	99.788	0.86
Lighter	19	3,484,166	0.05	99.821	0.89

Supplementary Table 6: Alignment statistics for GAGE chromosome 14 dataset using BWA-MEM.

Alignment statistics for the GAGE chr14 data set using BWA-MEM [4] v0.7.9a-r786 with default parameters.

		Read Level		Base Level	
	k	Mapped Reads	Increase (%)	Matches/aligned base (%)	Increase (%)
Original	-	36,493,776	-	97.093	-
Quake	19	32,560,823	-10.78	99.826	2.81
SOAPec	19	36,493,999	0.00	97.394	0.31
Musket	23	36,494,472	0.00	98.165	1.1
Bless	31	36,481,303	-0.03	98.661	1.61
Lighter	19	36,494,905	0.00	98.369	1.31

References

[1] M. Holtgrewe. Mason–a read simulator for second generation sequencing data. *Technical Report FU Berlin*, 2010.

[2] W. Huang, L. Li, J. R. Myers, and G. T. Marth. Art: a next-generation sequencing read simulator. *Bioinformatics*, 28(4):593–594, 2012.

[3] B. Langmead and S. L. Salzberg. Fast gapped-read alignment with bowtie 2. *Nature methods*, 9(4):357–359, 2012.

[4] H. Li. Aligning sequence reads, clone sequences and assembly contigs with bwa-mem. *arXiv preprint arXiv:1303.3997*, 2013.

[5] F. Putze, P. Sanders, and J. Singler. Cache-, hash-, and space-efficient bloom filters. *J. Exp. Algorithmics*, 14:4:4.4–4:4.18, Jan. 2010.
